# Personal, behavioral and socio-environmental predictors of overweight incidence in young adults: 10-yr longitudinal findings

**DOI:** 10.1186/1479-5868-10-37

**Published:** 2013-03-25

**Authors:** Virginia Quick, Melanie Wall, Nicole Larson, Jess Haines, Dianne Neumark-Sztainer

**Affiliations:** 1 Eunice Kennedy Shriver National Institute of Child Health and Human Development, Division of Epidemiology, Statistics and Prevention Research, NIH, DHHS, Bethesda, MD, 20892, USA; 2 Division of Epidemiology and Community Health, University of Minnesota, 1300 South Second Street, Suite 300, Minneapolis, MN, 55454, USA; 3 Division of Biostatistics, School of Public Health, University of Minnesota, 1300 South Second Street, Suite 300, Minneapolis, MN, 55454, USA; 4 Family Relations & Applied Nutrition, University of Guelph, 50 Stone Road East, Guelph, Ontario, N1G 2W1, Canada

**Keywords:** Adolescents, Young adults, Overweight, Weight control

## Abstract

**Background:**

The objective of this study was to identify 10-year longitudinal predictors of overweight incidence during the transition from adolescence to young adulthood.

**Methods:**

Data were from Project EAT (Eating and Activity in Teens and Young Adults). A diverse, population-based cohort (N = 2,134) completed baseline surveys in 1998–1999 (mean age = 15.0±1.6, ‘adolescence’) and follow-up surveys in 2008–2009 (mean age = 25.4±1.7, ‘young adulthood’). Surveys assessed personal, behavioral and socio-environmental factors hypothesized to be of relevance to obesity, in addition to height and weight. Multivariable logistic regression was used to estimate the adjusted odds for each personal, behavioral and socio-environmental factor at baseline, and 10-year changes for these factors, among non-overweight adolescents (n = 1,643) being predictive of the incidence of overweight (BMI ≥ 25) at 10-year follow-up.

**Results:**

At 10-year follow-up, 51% of young adults were overweight (26% increase from baseline). Among females and males, higher levels of body dissatisfaction, weight concerns, unhealthy weight control behaviors (e.g., fasting, purging), dieting, binge eating, weight-related teasing, and parental weight-related concerns and behaviors during adolescence and/or increases in these factors over the study period predicted the incidence of overweight at 10-year follow-up. Females with higher levels of whole grain intake and breakfast and dinner consumption frequency during adolescence were protected against becoming overweight. Among males, increases in vegetable intake protected against the incidence of overweight 10 years later.

**Conclusions:**

Findings suggest that obesity prevention interventions for adolescents should address weight-specific factors from within the domains of personal, behavioral, and socio-environmental factors such as promoting positive body image, decreasing unhealthy weight control behaviors, and limiting negative weight talk.

## Background

The high prevalence of obesity is of public health concern [1]. Research suggests that the growing prevalence of obesity over recent decades [2] is most likely due to a myriad of personal, behavioral and socio-environmental factors that, unlike genetic factors, are modifiable via public health interventions [3]. Furthermore, growing evidence suggests that events (e.g., experiencing weight teasing) during and throughout adolescence into young adulthood, a critical and sensitive time period for mental and physical growth, may influence obesity risk later in life [4]. In order to guide the development of obesity prevention interventions and policies, it is important to identify factors during adolescence and throughout the transition to young adulthood that have long-term implications for weight gain and the incidence of obesity.

Longitudinal studies that span the period from adolescence to young adulthood and comprehensively examine a number of personal, behavioral and socio-environmental factors predicting excess weight gain are lacking. Most longitudinal studies are short-term [5,6], and have included a limited number of predictors of overweight and obesity onset [7,8]. Existing evidence from these studies suggests that personal (e.g., weight concerns, depression), behavioral (e.g., weight control behaviors) and socio-environmental (e.g., decreased availability of healthy food) factors are associated with obesity [6,7,9] and may influence changes in weight status during this transitional period. For example, among adolescent girls (11–15 years) followed for over four years, depressive symptoms, weight control behaviors, and perceived parental obesity predicted obesity onset [7]. While these previous studies have provided important information, research that follows adolescents over a longer period of time into early adulthood and assesses a broad array of potential risk and protective factors using a theoretical framework is needed.

In Project EAT-II, the second wave of a population-based cohort study that followed adolescents over a 5-year period, variables found to predict overweight onset among both males and females were body dissatisfaction, weight concerns, skipping breakfast, use of unhealthy weight control behaviors and parental perceived concern about the child’s weight [10]. In general, factors found to predict overweight incidence were similar for both genders; however, fewer behavioral factors were found to be significantly associated with overweight in males as compared with females [10]. Given the many life events that may occur from adolescence to young adulthood (e.g., getting a job, moving out of parents’ home, entering long-term romantic relationships, attending college), it is of interest to explore whether similar factors during adolescence and the transition to adulthood predict the incidence of overweight status at 10-year follow-up [11]. Thus, the aim of this study was to identify personal, behavioral and socio-environmental factors during adolescence, and 10-year changes in these variables from adolescence to young adulthood, which predict the incidence of overweight in the Project EAT population-based cohort in order to better inform obesity interventions. It was hypothesized that factors similar to those found in the 5-year Project EAT follow-up study [10], including gender differences in behavioral factors, would predict overweight incidence 10 years later.

## Methods

### Study design

Project EAT (Eating and Activity in Teens and Young Adults) is a 10-year longitudinal study designed to examine factors associated with weight-related outcomes in a diverse sample of young people. The sample for the present study comprises 2,134 participants who responded to both baseline and 10-year follow-up surveys, provided complete height and weight data, and who were not pregnant at follow-up. At baseline, for Project EAT-I (Time 1), 4,746 junior and senior high school students (mean age = 15.0 ± 1.6) at 31 public schools in the Minneapolis/St. Paul metropolitan area of Minnesota completed classroom-administered surveys during the 1998–1999 academic year [12]. In 2008–2009, Project EAT-III participants (mean age = 25.4 ± 1.7**)** were asked to complete a follow-up survey online or by mail. Among those who could be contacted at the 10-year follow-up, the response rate was 66.4% (48.2% of the original school-based sample). The University of Minnesota’s Institutional Review Board approved all protocols used in Project EAT. Additional details of the study design have been reported elsewhere [13].

### Survey development and measures

Development of the Project EAT-I survey was guided by Social Cognitive Theory [14], focus groups with adolescents [15], an extensive literature review, content reviews by multi-disciplinary experts, and pilot testing. The Social Cognitive Theory proposes that personal, behavioral, and socio-environmental factors work in a dynamic and reciprocal fashion to influence health behavior [14]. Previous etiological research studies among youth have found that weight status is influenced by factors both at the individual and environmental levels [16,17]; thus, the Social Cognitive Theory is an appropriate framework for exploring factors that may increase the risk of weight gain over time. To allow for longitudinal comparisons, key items from the Project EAT-I survey were retained at EAT-III. Decisions to retain or drop items were based on the relevance of items to the current study aims, their use in earlier analyses, and the performance of represented constructs in the peer-reviewed literature. The EAT-I survey was not originally designed to assess predictors of physical activity, so several new items were added to the EAT-III survey reflecting the study’s broader ecological perspective with a greater focus on physical activity and its correlates [18]. EAT-III survey changes were made to ensure relevance to the study population as they were transitioning to more independent lifestyles and establishing new careers, households, and families as young adults [18]. A majority of items in the follow-up survey remained as they were in the original survey or with minor alterations such as a shortening a scale that did not reduce the Cronbach’s alpha or compromise content validity. Test-retest reliability over a 2-week period was assessed at baseline in a diverse sample of 161 adolescents [12] and at 10-year follow-up in a diverse sample of 66 young adults [18]. Test-retest reliabilities for items on the EAT-III survey were moderate to good and Cronbach’s alphas were >0.7 for 83% of developed scales [18]. Validity was not examined specifically for EAT-III but has been reported in previous work we have cited. Test-retest reliability coefficients for baseline survey measures are reported in this study.

### Measures

#### Outcome measure

Self-reported height and weight were used to calculate body mass index (BMI) [(weight(kg)/height(m^2^)] at Time 1 and 3. At Time 1, high correlations were found between self-reported and measured BMI in the sample of male (r = 0.88) and female (r = 0.85) adolescents [19]. Anthropometric measures were not completed in the full sample at Time 3; however, very high correlations between self-reported and measured BMI were found in a validation subsample of 63 male and 62 female EAT-III study participants (r = 0.95 for males and r = 0.98 for females). In some instances (n = 117) where self-reported BMI data at Time 1 were not available, but measured BMI data were available at Time 1, a single randomly imputed value of BMI was obtained from the multivariate normal expectation-maximization (EM) algorithm (PROC MI in SAS 9.2) utilizing measured BMI, age, gender, race/ethnicity, and socio-economic status as predictive information. A single imputed value was used and not multiple imputed values because there was a strong correlation between measured and self-reported BMI and very little variability of the imputed values. At Time 1, overweight status was determined based on a BMI at or above the 85th percentile for sex and age using reference data from the Centers for Disease Control and Prevention [20]. Weight status at Time 3 was defined according to current BMI guidelines for adults (overweight: BMI ≥ 25 kg/m^2^) [21].

#### Personal variables

Body satisfaction was measured using a modified version of the Body Shape Satisfaction Scale [22]; higher scores indicated higher body satisfaction (Cronbach’s α = 0.92; range: 10–50; test-retest: r = 0.68-0.77 for individual items). Depressive symptoms were assessed using a 6-item scale developed by Kandel and Davies [23]; higher scores indicated more severe depressive mood (Cronbach’s α = 0.82; range 10–30; test-retest r = 0.31-0.72 for individual items). Weight concerns were assessed by asking participants to indicate how strongly they agreed with the statements: a) “I think a lot about being thinner” (test-retest r = 0.78); and b) “I am worried about gaining weight” (test-retest r = 0.72). Higher summed responses indicated greater concern (Cronbach’s α = 0.81, range: 4–8).

#### Behavioral variables

Dietary intake (total energy and daily servings of fruit, vegetables, whole grains, and sugar-sweetened beverages) was assessed using the 2007 Willett semi-quantitative food frequency questionnaire (FFQ) at Time 3 [24] and the youth form of this questionnaire at Time 1 [25]. Prior studies have examined the reliability and validity of intake estimates based on these tools [24-27]. In addition, the comparability of estimates based on the two FFQs was examined in a subsample of 91 male and 103 female participants in EAT-III who completed both questionnaires [28]. Fast food consumption (test-retest r = 0.46, range: 0–10) was assessed with the question: “In the past week, how often did you eat something from a fast-food restaurant (like McDonald’s, Burger King, Hardee’s, etc.)?” Frequency of eating breakfast (test-retest r = 0.77), lunch (test-retest r = 0.71), and dinner (test-retest r = 0.72) were self-reported for the past week [29].

Moderate-to-vigorous physical activity was assessed using two items adapted from the modified Leisure Time Exercise Questionnaire [30]. Participants were asked to separately report how many hours they engaged in moderate activities (e.g., walking quickly; test-retest r = 0.52) and strenuous activities (e.g., biking fast; test-retest r = 0.63) in a usual week; total weekly hours were computed (possible range: 0–16). Sedentary behaviors were assessed using items that separately asked about hours of television/video/DVD watching and computer use on an average weekday and weekend day (test-retest r = 0.66-0.80 for individual items); total weekly hours of sedentary behavior were computed (possible range: 0–105 at Time 1 and 0–126 at Time 3) [31]. Score ranges of sedentary behaviors at Time 1 and 3 differed because an additional measure of video/electronic game use was included in the follow-up survey. Additionally, the age-appropriate measure used to assess leisure-time computer use was worded differently at baseline (using a computer [not for homework]) than at follow-up (using a computer [not for work or school]).

Use of unhealthy and extreme weight control behaviors used in the past year were assessed by asking participants whether they had used (yes/no) any of five unhealthy methods (e.g., skipped meals) or any of four extreme methods (e.g., used laxatives). For the majority of specific weight control behaviors used in the past year, test-retest Kappa’s ranged from 0.50 to 0.68; however, lower values were found for laxatives (ĸ = 0.29) and food substitutes (ĸ = 0.44).

Binge eating was assessed with two questions: “In the past year, have you ever eaten so much food in a short period of time that you would be embarrassed if others saw you (binge-eating)?” and “During the times when you ate this way, did you feel you couldn’t stop eating or control what or how much you were eating?” (yes/no for each question; test-retest ĸ = 0.64 [first question] and 0.23 [second question]). Dieting was assessed by asking “How often have you gone on a diet during the last year? By ‘diet’ we mean changing the way you eat so you can lose weight.” (test-retest r = 0.71). As in past analyses [10], responses were dichotomized to identify non-dieters (never) and dieters (one or more times).

#### Socio-environmental variables

Home availability of healthful foods (3-items; range: 3–12; test-retest r = 0.56-0.59 for individual items) and low-nutrient, high-caloric snack foods (4-items; range: 4–16; test-retest r = 0.55-0.72 for individual items) were self-reported. Parental weight-related concerns and behaviors were based on agreement with four items assessing perceptions of whether one’s mother and father encouraged them to diet or dieted themselves to lose or maintain weight (Cronbach’s α = 0.78; range: 4–16; test-retest r = 0.58-0.64 for individual items). Weight-related teasing was assessed with the question, “How often did any of the following things happen to you: You are teased about your weight?” Participant responses were never, less than once a year, a few times a year, a few times a month and at least once a week. Response categories “never” and at least a few times per month or more of being teased about your weight in the past year were dichotomized into no/yes categories, respectively, for ease of analysis. Peer dieting behaviors were assessed with the question, “Many of my friends diet to lose weight or keep from gaining weight.” (test-retest r = 0.48); participants were classified as having friends that dieted if they reported any such behavior (yes/no). Perceived overweight status for one’s biological mother (test-retest r = 0.83) and father (test-retest r = 0.83) was based on adolescent report; participants were classified as having overweight parents if they reported that at least one parent was “overweight” or “very overweight.”

#### Demographic variables

Participant gender, age, ethnic/racial identity, and socioeconomic status (SES) were self-reported at baseline. SES was based on several variables reported at baseline, including the highest education level completed by either parent, eligibility for public assistance, eligibility for free or reduced-cost school meals, and parental employment status [32].

### Data analyses

The prevalence of overweight status was calculated by demographics for the sample at Time 1 (baseline) and Time 3 (follow-up). All further analyses focused only on those individuals who were not overweight at baseline (n = 887 female and n = 756 males) in order to identify predictors of incidence of overweight status. Descriptive means or proportions of all the personal, behavioral, and socio-environmental predictor variables at Time 1 and changes in them from Time 1 to Time 3 were calculated. Tests of differences between females and males found 19 of the 24 Time 1 predictors to be significantly different across gender (results not shown, the five predictors that were not significantly different were fruit servings, vegetable servings, home availability of healthful foods, home availability of high-caloric snack foods, and parental weight concern). This finding, in addition to the finding of a statistically significant difference in incident overweight status at Time 3 by gender led us to conduct all further analyses stratified by gender. Paired t-tests were used to test for mean changes in the predictor variables from Time 1 to Time 3 stratified by gender. In addition, Pearson correlations between Time 1 and Time 3 predictor variables were used to quantify stability. Incidence of overweight at follow-up was modeled using gender-stratified logistic regression models including each of the personal, behavioral, or socio-environmental predictor variables, controlling for age, SES, and race/ethnicity. For each predictor variable, two separate regressions were fit: one including only the Time 1 predictor and the other including both the Time 1 predictor and the 10-year change (Time 3 – Time 1) in the predictor. Odds ratios from models including only the Time 1 predictor represent the overall increased prospective odds of becoming overweight associated with a one unit difference in the Time 1 predictor, regardless of how the predictor changed over time. For the models that additionally included 10-year change in the predictor, the estimate for the change predictor represents the increased odds of becoming overweight associated with a one unit change in the predictor over the 10 years when comparing individuals who were identical on the predictor at Time 1. Thus, we reported the odds ratio from the first model corresponding to the baseline predictor and the odds ratio from the second model corresponding to the 10-year change in the predictor (where the baseline predictor is controlled). Additionally, to determine whether there was a differential effect of change in the predictor on incident overweight dependent on baseline predictor values, we also conducted regressions including interactions between the baseline predictor and change variables. Results from post-hoc investigation of interactions between baseline and change predictors did not find any significant interaction effects for males nor females on overweight incident status using Bonferonni correction. Thus, the effects from 10-year changes in the predictors over time are the same regardless of where a participant started at baseline. Total energy intake was included in analysis of FFQ-derived dietary predictor variables to help account for measurement error in dietary assessment [33]. Regressions excluded individuals who did not have a self-reported or measured height and weight data at baseline or follow-up (n = 63), and excluded women who were pregnant at follow-up (n = 90).

Analyses were weighted using the response propensity method to account for differential loss to follow-up. Response propensities were estimated using a logistic regression of response at follow-up on a large number of predictor variables from the Project EAT-I survey [34]. Weights were also calibrated so that the weighted total sample sizes used in analyses for each gender cohort accurately reflect the actual observed sample sizes in those groups. The weighting method resulted in estimates representative of the demographic make-up of the original school-based sample, thereby allowing results to be more fully generalizable to the population of young people in the Minneapolis/St. Paul metropolitan area. Specifically, with regard to ethnicity/race, the weighted sample was 48% white, 20% African American, 18% Asian, 5% Hispanic, 3% Native American, 5% mixed or other race/ethnicity. All analyses were conducted in SAS software (version 9.2, 2003; SAS, Inc., Cary, NC) in 2012. Statistical significance was set at alpha < 0.05.

## Results

### Prevalence of overweight and change over time in predictors

At baseline, 25.1% of females and 25.9% of males were overweight, while at follow-up, 47.5% of females and 56.1% of males were overweight; age-appropriate categorizations were used at the two time points (Table 1). Among the non-overweight adolescents at baseline, 34.2% of the females and 45.4% of the males became overweight by young adulthood 10 years later, using age-appropriate definitions.

**Table 1:** **Prevalence of overweight status in adolescents and young adults in Project EAT**^‡^

**Characteristic**	**Time 1**	**Time 3**	**Incidence of Overweight at Time 3**^**a**^
	**Year 1999**	**Year 2009**	
	Overweight*	**Overweight**	
	**% (N)**^†^	**% (N)**	**% (N)**
**All Females (N = 1133)**	25.1 (285)	47.5 (538)	34.2 (304)
**Baseline school level**			
Middle School cohort (n = 325)	31.2 (101)	47.3 (154)	32.9 (77)
High School cohort (n = 808)	22.7 (183)	47.5 (384)	34.7 (227)
**Race/ethnicity**			
White (n = 541)	19.6 (106)	42.5 (230)	31.6 (144)
Black (n = 231)	36.3 (84)	61.9 (143)	47.2 (73)
Hispanic (n = 60)	30.84 (19)	50.5 (30)	30.5 (13)
Asian (n = 201)	20.3 (41)	41.6 (83)	31.5 (53)
Native American (n = 37)	40.1 (15)	51.7 (19)	34.0 (8)
Multi-racial/other (n = 63)	32.5 (20)	50.9 (32)	29.9 (13)
**Socioeconomic Status**			
Low (n = 191)	29.8 (57)	58.3 (112)	44.4 (62)
Low-middle (n = 203)	31.0 (63)	52.4 (106)	37.6 (55)
Middle (n = 301)	26.4 (79)	52.5 (158)	39.6 (92)
Middle-high (n = 254)	22.4 (57)	41.8 (106)	29.6 (61)
High (n = 154)	16.5 (25)	32.9 (51)	22.7 (31)
**All Males (N = 1001)**	25.9 (245)	56.1 (561)	45.4 (343)
**Baseline school level**			
Middle School cohort (n = 295)	29.2 (86)	49.5 (146)	36.5 (78)
High School cohort (n = 706)	24.5 (173)	58.8 (415)	48.9 (266)
**Race/ethnicity**			
White (n = 510)	24.4 (125)	53.0 (271)	42.2 (166)
Black (n = 145)	23.6 (34)	47.7 (69)	35.6 (40)
Hispanic (n = 68)	35.6 (24)	64.6 (44)	54.3 (24)
Asian (n = 192)	24.6 (47)	66.0 (127)	58.9 (87)
Native American (n = 32)	41.8 (13)	59.3 (19)	35.3 (7)
Multi-racial/other (n = 53)	29.3 (16)	59.0 (32)	50.0 (19)
**Socioeconomic Status**			
Low (n = 159)	31.0 (49)	70.9 (113)	61.8 (69)
Low-middle (n = 187)	28.3 (53)	64.3 (120)	54.8 (75)
Middle (n = 239)	22.0 (57)	52.6 (126)	42.5 (79)
Middle-high (n = 247)	24.3 (60)	49.5 (122)	39.2 (75)
High (n = 132)	21.6 (29)	43.0 (57)	32.1 (34)

*Overweight defined as BMI above the 85th percentile for adolescents at Time 1 [20] and BMI ≥ 25 for young adults at Time 3 [21].

^a^Incidence is based on individuals not overweight at Time 1 (n = 887 females, n = 756 males).

^†^All percents (%) and n’s are weighted with non-response propensity weights.

^‡^Age (years) for adolescents (15.0 ± 1.6) and young adults (25.4 ± 1.7).

Table 2 presents means and percentages for continuous and categorical baseline predictor variables and changes in these predictor variables from baseline to 10-year follow-up. For personal factors, body satisfaction decreased while depression and weight concerns increased significantly from baseline to follow-up among females and males. For behavioral factors, females and males had significant increases in vegetable and whole grain intake and decreases in sugar-sweetened beverage intake from baseline to follow-up. Males significantly decreased their frequency of meals (i.e., breakfast and lunch) and increased their fast food consumption from baseline to follow-up; in contrast, females increased their frequency of meals (i.e., breakfast, lunch and dinner) and decreased their fast food consumption from baseline to follow-up. Additionally, extreme weight control behaviors, dieting and binge eating increased significantly from baseline to follow-up among females and males. For socio-environmental factors, home availability of healthful foods and high-caloric snack foods decreased, while peer dieting behaviors increased significantly from baseline to follow-up among females and males.

**Table 2:** Non-overweight adolescents: Baseline (Time 1) and changes in personal, behavioral, and socio-environmental factors

		**Females (N = 887)**		**Males (N = 756)**	
**Variable**	**Possible scale ranges**	**Variables at Time 1**	**Change in variables (Time 1 to Time 3)**	**Variables at Time 1**	**Change in variables (Time 1 to Time 3)**
		**Mean ± SD or %**	**Change in Mean or % (95% CI)**	**Mean ± SD or %**	**Change in Mean or % (95% CI)**
**Personal Factors**					
Body satisfaction	10-50	33.7 ± 8.7	−2.99 (−3.69, -2.30)*	38.2 ± 8.1	−2.12 (−2.87, -1.36)*
Depressive symptoms	10-30	18.4 ± 4.4	**0.52 (0.13, 0.91)**	16.1 ± 4.3	**1.23 (0.82, 1.64)**
Weight concerns	4-8	2.6 ± 0.9	**0.33 (0.26, 0.40)**	1.9 ± 0.8	**0.28 (0.20, 0.36)**
**Behavioral Factors**					
Eating Behaviors					
Energy Intake (kcal)	--	1941.4 ± 834.8	**104 (31.1, 177)**	2271.1 ± 1127.1	1.20 (−103, 105)
Fruits servings (servings/d)	--	2.2 ± 1.7	−0.02 (−0.18, 0.14)	2.2 ± 1.6	**−0.34 (−0.50, -0.18)**
Vegetable servings (serving/d)	--	1.8 ± 1.3	**1.31 (1.15, 1.48)**	1.6 ± 1.4	**0.97 (0.80, 1.15)**
Whole grain intake (servings/d)	--	0.9 ± 0.8	**1.11 (1.01, 1.22)**	1.1 ± 0.9	**0.94 (0.78, 1.10)**
Sugar-sweetened beverages (serving/d)	--	1.0 ± 0.9	**−0.30 (−0.39, -0.22)**	1.3 ± 0.9	**−0.18 (−0.30, -0.05)**
Fast food (times/week)	--	1.6 ± 1.6	**−0.31 (−0.44, -0.18)**	1.8 ± 1.6	**0.27 (0.09, 0.45)**
Breakfast (times/week)	--	3.7 ± 2.5	**0.62 (0.42, 0.82)**	4.3 ± 2.6	**−0.76 (−1.00, -0.53)**
Lunch (times/week)	--	5.3 ± 2.0	**0.32 (0.16, 0.48)**	6.0 ± 1.8	**−0.38 (−0.54, -0.21)**
Dinner (times/week)	--	5.9 ± 1.7	**0.40 (0.26, 0.53)**	6.4 ± 1.2	−0.07 (−0.19, 0.05)
Weight Control Behaviors (WCBs)					
Any Unhealthy WCBs (% yes)	--	51.5	1.53 (−2.55, 5.61)	26.0	−0.29 (−4.33, 3.76)
Extreme WCBs (% yes)	--	9.5	**8.42 (5.40, 11.4)**	2.4	**2.54 (0.71, 4.37)**
Any Dieting (% yes)	--	49.1	**5.84 (1.72, 9.97)**	15.7	**9.53 (5.79, 13.3)**
Binge eating (% yes)	--	8.6	**4.60 (1.85, 7.34)**	2.4	**2.69 (0.87, 4.52)**
Physical Activity					
Moderate and vigorous (hrs/wk)	0-16	5.9 ± 4.4	**−2.33 (−2.68, -1.98)**	7.6 ± 4.6	−**2.62 (−3.04, -2.01)**
Sedentary behaviors (hrs/wk)	0-105^†^	39.7 ± 17.3	**−8.55 (−10.1, -6.96)**	43.5 ± 21.4	−0.68 (−3.00, 1.62)
**Socio-Environmental Factors**					
Home availability of healthful foods	3-12	9.7 ± 1.8	**−0.67 (−0.82, -0.52)**	9.6 ± 1.8	**−0.79 (−0.96, -0.61)**
Home availability of high-caloric snack foods	4-16	11.0 ± 2.8	**−1.13 (−1.35, -0.90)**	11.1 ± 2.6	**−0.77 (−1.01, -0.52)**
Parental weight-related concerns and behaviors^A^	4-16	6.5 ± 2.6	**---**	6.7 ± 2.8	**---**
Weight-related teasing (% yes)	--	39.1	0.22 (−3.88, 4.32)	26.4	**10.4 (5.98, 14.8)**
Peer dieting behaviors (% yes)	--	72.9	**13.8 (10.2, 17.5)**	39.3	**26.4 (21.0, 31.7)**
Perceived parental overweight (% yes)^A^	--	41.5	**---**	44.9	**---**

SD = standard deviation; CI = confidence interval.

*Boldface indicates statistical significant (p < 0.05) change from Time 1 to Time 3.

^A^Variables were not assessed at 10-year follow-up (Time 3) so changes were not determined.

^†^Possible score range for sedentary behaviors at Time 3 is 0–126 hrs/wk.

The correlation (stability) from Time 1 to Time 3 was positive for all predictors and significantly different from zero albeit generally low in magnitude. Across all 23 predictors with Time 3 measures, the average correlation for males was 0.16 and for females was 0.23. The highest stability in predictive factors among both males and females was observed for personal factors (i.e., body satisfaction: r (males) = 0.25, r (females) = 0.33; depressive symptoms: r (males) = 0.26, r (females) = 0.22; and weight concerns: r (males) = 0.25, r (females) = 0.34); the average Time 1 to Time 3 correlation for personal factors was approximately 0.25 in males and 0.33 in females.

### Females: baseline personal, behavioral and socio-environmental factors, and associations with incidence of overweight status

**Figure 1:**
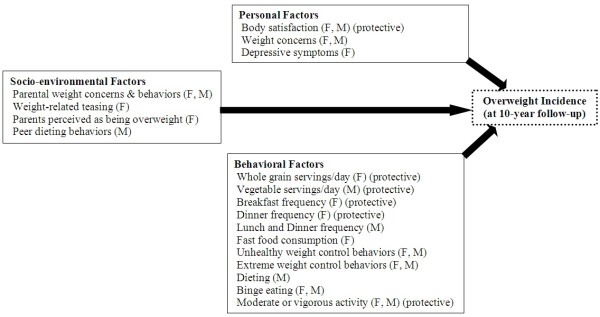
**Factors during adolescence or 10-year changes in factors from adolescence to young adulthood found to predict the incidence of overweight at 10-year follow-up#†. **^#^All factors are risk factors unless specified as protective. ^†^Factors are included that significantly predicted the incidence of overweight at follow-up or increases of these factors over the 10-year study period in either females (F) or males (M).

Among female participants who were not overweight at baseline, several personal, behavioral and socio-environmental factors assessed at baseline during adolescence significantly predicted the incidence of overweight (i.e., the odds of non-overweight adolescents becoming overweight) 10 years later (Table 3 and Figure 1). For personal factors, higher body satisfaction during adolescence predicted lower incidence of overweight (OR = 0.96, CI_95_ = 0.95-0.98) and greater weight concerns during adolescence predicted a higher incidence of overweight at follow-up (OR = 1.45, CI_95_ = 1.23-1.71). Of the behavioral factors assessed, use of unhealthy weight control behaviors during adolescence predicted overweight incidence (OR = 1.76, CI_95_ = 1.29-2.41). Additionally, consuming more whole grains (OR = 0.71, CI_95_ = 0.54-0.93) and eating breakfast (OR = 0.91, CI_95_ = 0.86-0.97) and dinner (OR = 0.88, CI_95_ = 0.81-0.95) more frequently at baseline predicted lower overweight incidence. Of the socio-environmental factors assessed, weight-related teasing experienced as an adolescent strongly predicted overweight incidence (OR = 1.66, CI_95_ = 1.21-2.27). Parental weight-related concerns and behaviors during adolescence also predicted overweight incidence (OR = 1.12, CI_95_ = 1.06-1.18). Additionally, females who perceived their biological parents as being overweight (perceived parental overweight) during adolescence were at risk for overweight incidence (OR = 1.41, CI_95_ = 1.01-1.98).

**Table 3:** 10-year longitudinal personal, behavioral, and socio-environmental predictors of incidence of overweight status by gender

	**Females**		**Males**	
	Incidence of overweight (N = 887)*		Incidence of overweight (N = 756)*	
**Variable**	**Time 1 Predictor [OR (95% CI)]**^**†**^	**10-year Changes in Predictor [OR (95% CI)]**^**‡**^	**Time 1 Predictor [OR (95% CI)]**^**†**^	**10-year Changes in Predictor [OR (95% CI)]**^**‡**^
**Personal Factors**				
Body satisfaction	**0.96 (0.95, 0.98)**^§^	**0.90 (0.88, 0.92)**	1.01 (0.99, 1.03)	**0.94 (0.92, 0.96)**
Depressive symptoms	1.00 (0.97, 1.04)	**1.05 (1.02, 1.09)**	1.02 (0.98, 1.05)	1.01 (0.98, 1.05)
Weight concerns	**1.45 (1.23, 1.71)**	**2.69 (2.15, 3.35)**	**1.49 (1.22, 1.82)**	**3.33 (2.69, 4.11)**
**Behavioral Factors**				
Eating Behaviors				
Energy Intake (kcal)^	0.91 (0.76, 1.09)	0.98 (0.80,1.20)	1.26 (0.98, 1.30)	0.99 (0.82,1.21)
Fruits servings (servings/d)^¶^	0.98 (0.87, 1.10)	0.98 (0.89, 1.09)	1.03 (0.92, 1.17)	1.04 (0.91, 1.18)
Vegetable servings (serving/d)^¶^	1.03 (0.90, 1.18)	1.03 (0.94, 1.12)	1.12 (0.97, 1.29)	**0.88 (0.78, 0.99)**
Whole grain intake (servings/d)^¶^	**0.71 (0.54, 0.93)**	0.93 (0.82, 1.07)	0.96 (0.78, 1.19)	1.01 (0.92, 1.12)
Sugar-sweetened beverages (serving/d)^¶^	1.14 (0.95, 1.38)	1.09 (0.93, 1.27)	1.09 (0.91, 1.31)	1.06 (0.94, 1.21)
Fast food (times/week)	0.95 (0.87, 1.04)	**1.15 (1.04, 1.27)**	1.02 (0.93, 1.12)	1.02 (0.95, 1.10)
Breakfast (times/week)	**0.91 (0.86, 0.97)**	0.97 (0.91, 1.04)	0.95 (0.90, 1.01)	1.02 (0.95, 1.09)
Lunch (times/week)	0.94 (0.87, 1.01)	1.00 (0.92, 1.09)	0.99 (0.91, 1.07)	**1.13 (1.03, 1.23)**
Dinner (times/week)	**0.88 (0.81, 0.95)**	0.94 (0.84, 1.05)	1.02 (0.90, 1.16)	**1.14 (1.00, 1.29)**
Weight Control Behaviors (WCBs)				
Unhealthy WCBs (yes/no)	**1.76 (1.29, 2.41)**	**2.12 (1.54, 2.91)**	1.32 (0.92, 1.89)	**3.28 (2.26, 4.78)**
Extreme WCBs (yes/no)	0.93 (0.55, 1.56)	**2.02 (1.39, 2.95)**	0.56 (0.20, 1.56)	**2.94 (1.37, 6.31)**
Dieting (yes/no)	1.29 (0.95, 1.74)	**3.01 (2.17, 4.17)**	**2.01(1.31,3.08)**	**3.25 (2.24, 4.72)**
Binge eating (yes/no)	1.31 (0.78, 2.20)	**2.27 (1.48, 3.49)**	**4.02 (1.11, 14.6)**	**2.84 (1.32, 6.09)**
Physical Activity				
Moderate and vigorous (hrs/wk)	1.00 (0.97, 1.03)	**0.95 (0.91, 0.99)**	1.03 (0.99, 1.06)	**0.96 (0.92, 1.00)**
Sedentary behaviors (hrs/wk)	1.01 (1.00, 1.01)	1.01 (1.00, 1.01)	0.99 (0.99, 1.00)	1.00 (1.00,1.01)
**Socio-environmental Factors**				
Home availability of healthful foods	0.97 (0.89, 1.05)	0.97 (0.89, 1.05)	1.05 (0.96, 1.14)	1.03 (0.95, 1.12)
Home availability of high-caloric snack foods	0.99 (0.94, 1.04)	1.05 (0.99, 1.11)	1.02 (0.96, 1.09)	1.05 (0.99, 1.11)
Parental weight-related concerns and behaviors^A^	**1.12 (1.06, 1.18)**	**---**	**1.10 (1.04, 1.16)**	---
Weight-related teasing (yes/no)	**1.66 (1.21, 2.27)**	**2.43 (1.75, 3.36)**	0.78 (0.55, 1.12)	**1.67 (1.20, 2.32)**
Peer dieting behaviors (yes/no)	1.01 (0.69, 1.46)	1.39 (0.82, 2.35)	1.50 (1.05, 2.13)	**2.29 (1.54, 3.40)**
Perceived parental overweight (yes/no)^A^	**1.41 (1.01, 1.98)**	**---**	1.21 (0.88, 1.67)	---

OR = odds ratio; CI = confidence interval.

*Analysis restricted to non-overweight participants at Time 1 (BMI percentile ≤85^th^).

^†^Adjusted for age, socioeconomic status (SES), and race/ethnicity.

^‡^Adjusted for age, socioeconomic status (SES), and race/ethnicity and Time 1 predictor variable.

^§^Boldface indicates significance at p < 0.05 level.

^Calories scaled by a 1,000 for ease of interpretation.

^¶^Additionally adjusted for energy intake.

^A^Variables were not assessed at Time 3 so change differences were not determined.

### Females: 10-year changes in personal, behavioral and socio-environmental factors, and associations with incidence of overweight status

Many personal, behavioral and socio-environmental factors that changed from adolescence to young adulthood were associated with the incidence of overweight at follow-up among females (see Table 3). Of the personal factors assessed, increased weight concerns and depressive symptoms from adolescence to young adulthood predicted higher overweight incidence (weight concerns: OR = 2.69, CI_95_ = 2.15-3.35; depressive symptoms: OR = 1.05, CI_95_ = 1.02-1.09), while 10-year increases in body satisfaction were protective against overweight incidence (OR = 0.90, CI_95_ = 0.88-0.92). Increases in weight control behaviors from adolescence to young adulthood were all associated with overweight incidence. For example, females who increased their use of unhealthy and extreme weight control behaviors, binge eating, and/or dieting (i.e. who went from not engaging in these behaviors at baseline to using them at follow-up) were two times at increased odds of becoming overweight at follow-up. Additionally, increases in fast food consumption from adolescence to young adulthood predicted overweight incidence (OR = 1.15, CI_95_ = 1.04-1.27). Increased weight-related teasing from adolescence to young adulthood was the only socio-environmental factor associated with overweight incidence (OR = 2.43, CI_95_ = 1.75-3.36).

### Males: baseline personal, behavioral, socio-environmental factors and associations with incidence of overweight status

Results of analyses examining the associations between baseline factors and incidence of overweight status in male participants were similar to those found among females, although fewer associations were statistically significant (see Table 3 and Figure 1). A higher level of weight concerns during adolescence was the only personal factor found to predict overweight incidence (OR = 1.49, CI_95_ = 1.22-1.82) in males. Of the behavioral factors assessed, dieting and binge eating during adolescence predicted a higher overweight incidence (dieting: OR = 2.01, CI_95_ = 1.31-3.08; binge eating: OR = 4.02, CI_95_ = 1.11-14.6). No other dietary intake variables or eating behaviors during adolescence predicted overweight incidence. The only socio-environmental factor during adolescence that predicted overweight incidence in males was parental weight-related concerns and behaviors (OR = 1.10, CI_95_ = 1.04-1.16).

### Males: 10-year changes in personal, behavioral, and socio-environmental factors and associations with incidence of overweight status

Similar to the findings among females, many personal, behavioral and socio-environmental factors that changed from adolescence to young adulthood were associated with the incidence of overweight at 10-year follow-up among males (see Table 3). Of the personal factors assessed, increased weight concerns from adolescence to young adulthood predicted overweight incidence (weight concerns: OR = 3.33, CI_95_ = 2.69-4.11) while increased body satisfaction over time predicted lower overweight incidence (OR = 0.94, CI_95_ = 0.92-0.96). Increases in weight control behaviors from adolescence to young adulthood were associated with overweight incidence. For instance, males with 10-year increases in unhealthy and extreme weight control behaviors, binge eating, and/or dieting (i.e. who went from not engaging in these behaviors at baseline to using them at follow-up) were at nearly three times the odds of becoming overweight at follow-up. Increased frequency of lunch and dinner consumption from adolescence to young adulthood predicted higher overweight incidence (lunch: OR = 1.13, CI_95_ = 1.03-1.23; dinner: OR = 1.14, CI_95_ = 1.00-1.29), while increases in vegetable servings was protective against overweight incidence (OR = 0.88, CI_95_ = 0.78-0.99). Increased weight-related teasing and peer dieting behaviors from adolescence to young adulthood were the only socio-environmental factors that predicted overweight incidence (weight-related teasing: OR = 1.67, CI_95_ = 1.20-2.32; peer dieting: OR = 2.29, CI_95_ = 1.54-3.40).

## Discussion

This study identified a number of personal, behavioral and socio-environmental factors during adolescence, and changes in these factors from adolescence to young adulthood, that are predictive of the incidence of overweight at 10-year follow-up. Among females and males higher levels of body dissatisfaction, weight concerns, unhealthy weight control behaviors (e.g., fasting), dieting, binge eating, weight-related teasing, and parental weight-related concerns and behaviors during adolescence, and/or increases in these factors over the 10-year study period predicted the incidence of overweight. Only a few healthy dietary behaviors (i.e., increased whole grain and vegetable intake) or eating patterns (e.g., eating breakfast on a more regular basis) were found to decrease the odds of becoming overweight 10 years later. For instance, among female participants, increased whole grain intake and regular consumption of breakfast and dinner during adolescence protected against the incidence of overweight, while increases in fast food consumption over the 10-year study period increased their odds of becoming overweight. These findings suggest the importance of intervening early and preventing the endorsement or progression of unhealthy behaviors during the adolescent years, by helping young people feel better about their bodies and avoiding unhealthy weight control behaviors, and by working with parents to provide a home environment in which conversations about weight, in particular weight-related teasing, are minimized.

There were some interesting gender-specific associations between weight-related variables and the incidence of overweight in the 10-year change and baseline models that have important implications for the timing of interventions. For instance, among females, endorsing unhealthy weight control behaviors during adolescence (baseline) and 10-year changes in these behaviors (either starting or continued use of unhealthy weight control behaviors) over time were both predictive of overweight incidence. Among males, 10-year changes in unhealthy weight control behaviors significantly predicted overweight incidence but endorsement of unhealthy weight control behaviors during adolescence was not predictive of overweight incidence in young adulthood. These findings among males may indicate that we are unable to predict if male adolescents will be become overweight based on their adolescent unhealthy weight control behaviors; instead what matters is where these males are ending up with endorsement of these behaviors as a young adult. These findings indicate that regardless of gender, initiating the endorsement of unhealthy weight control behaviors will place individuals at increased risk for weight gain and odds for becoming overweight as a young adult compared to those who never endorse these behaviors, and discontinuing these behaviors during the transition from adolescence to young adulthood may help to prevent the onset of overweight.

Our findings build on previous shorter-term studies showing that weight concerns [35], body dissatisfaction [36], and unhealthy weight control behaviors [8,37] during adolescence increase risk for weight gain over time. Adolescents with high levels of weight concerns and body dissatisfaction may use weight control behaviors, such as dieting, as a means to reach their ideal body weight or shape [38]. However, dieting and the use of other restrictive, unhealthy weight control behaviors (e.g., fasting, taking laxatives) may lead to overeating or binge eating and result in weight gain [39]. For instance, a 4-year longitudinal study of children and early adolescents (ages 6–12) found binge eating and dieting to be strongly predictive of increases in body fat; children who reported binge eating gained, on average, 15% more fat mass compared to children who did not report binge eating [8]. Thus, findings suggest that unhealthy weight control behaviors are ineffective and potentially harmful strategies. Support is needed to help adolescents adopt healthier eating behaviors that emphasize a non-dieting approach to weight management and to be more accepting of their bodies.

Previous research examining dietary variables have found significant associations between measures of dietary intake such as sugar-sweetened beverage intakes and body weight [40,41] although findings are not always consistent nor strong in magnitude [40]. In the current study, few dietary intake behaviors during adolescence were consistently found across analyses and gender to be predictive of the incidence of overweight 10 years later. When interpreting these results, it is important to consider the methodological challenges in the assessment of dietary intake, particularly the assessment of energy intake. Although analyses of dietary variables in this study were adjusted for caloric intake to help account for reporting bias, residual confounding may still be present, and there may be differences by weight status [42]. Additionally, there were challenges in examining 10-year changes in dietary intake; however, age-appropriate food frequency questionnaires developed along similar lines were utilized [28]. Nevertheless, findings from the present study indicate that some healthy eating behaviors (i.e., increased whole grain and vegetable intake) during adolescence are protective against overweight incidence in young adulthood and need to be considered as important aspects in obesity prevention and treatment interventions.

Meal frequency findings were inconsistent between female and male adolescents, but suggest that female adolescents who frequently consume breakfast and dinner are protected against excessive weight gain in young adulthood, which supports other prospective [43,44] and cross-sectional studies [45]. Unexpectedly, among male adolescents, increases in the frequency of lunch and dinner during the transition from adolescence to young adulthood increased their risk of becoming overweight 10 years later. Over this same time period, male adolescents increased their fast food consumption; thus, these additional meals consumed by male adolescents were potentially unhealthy food choices that led to weight gain and obesity risk. These findings suggest the importance of encouraging male adolescents to make healthy meal choices.

Higher levels of weight-related teasing and parental weight-related concerns and behaviors during adolescence and, in some instances, increases in these factors from adolescence to young adulthood were found to be predictive of incidence of overweight 10 years later. These findings suggest that parental weight-related comments and pressures to lose weight during adolescence may be harmful and should be discouraged. Research has shown weight-related teasing during adolescence to be associated with disordered eating [46] and poor psychological well-being [47] and, as found in the present study, predictive of overweight risk. Thus, obesity prevention and treatment interventions should also involve educating adolescents and their surrounding network of families and friends about the importance of avoiding negative weight talk and strategies to promote positive, supportive conversations. Findings from this study were comparable to the 5-year longitudinal study findings [10], further supporting the notion that socio-environmental factors experienced during adolescence influence weight status into young adulthood.

Several strengths of the current study enhance the utility of the findings. First, because this was a prospective study, there was reduced chance of recall bias. Second, this study was conducted in a large, ethnically/racially and socio-economically diverse population that was similar in terms of racial/ethnic composition to the U.S. population of adolescents and youth adults [48], improving our ability to generalize the findings. Third, variable selection was guided by Social Cognitive Theory and a myriad of personal, behavioral and socio-environmental factors were assessed, allowing for a comprehensive examination of potential predictors of overweight incidence.

It is also important to take study limitations into consideration when interpreting the findings. The attrition of participants and use of self-reported BMI and dietary intake data may have introduced bias to the study findings. Compared with the original sample, adolescents who completed both the baseline and follow-up survey were more likely to be female, white and in the upper SES categories. However, sampling weights correcting for non-response bias were used in all analyses. Additionally, high correlations between self-reported and measured BMI values were found at Time 1 [19] and Time 3 [37]. Due to the limited number of physical activity measures assessed at Time 1, we were unable to adequately address factors such as perceived barriers to physical activity, social support for physical activity, and physical activity self-efficacy in terms of their ability to predict overweight incidence in this study. More comprehensive measures of these potential predictors of physical activity were added later in study waves. Future research will be needed to more thoroughly examine factors relevant to energy expenditure versus energy intake. In addition, we were unable to fully explore the role of life events that commonly occur during the transition to young adulthood such as finishing school, moving out of parent’s house, marriage and starting a full-time job; in future studies, markers of transition to young adulthood, and their timing, should be measured and examined in relation to weight changes overtime. Also, when interpreting our 10-year change findings, it is important to consider that we are unable to determine whether the change of certain behaviors or attitudes occurred before an individual became overweight over the 10-year time period. Thus, we are unable to rule out the possibility of reverse causation. Testing for interactions between personal, behavioral and socio-environmental factors that could further help to determine how these factors interact with one another to influence weight gain among adolescents was beyond the scope of this study, but could be considered in future analyses.

## Conclusions

Study findings suggest that a number of personal, behavioral and socio-environmental factors during adolescence and changes in these factors throughout the transition from adolescence to young adulthood influence risk for becoming overweight in young adulthood. In particular among females and males, higher levels of body dissatisfaction, unhealthy weight control behaviors, weight concerns, dieting, binge eating, weight-related teasing, and/or parental weight-related concerns and behaviors during adolescence or 10-year increases in these factors predicted the incidence of overweight at follow-up. Findings suggest that the Social Cognitive Theory may be an appropriate theoretical framework for guiding the development of individual-level or family-based obesity prevention interventions, given that factors from within each of the domains of personal, behavioral, and socio-environmental factors contributed to explaining overweight incidence over a 10-year period. Findings also suggest that obesity prevention and treatment interventions for young people might benefit from a non-dieting approach to healthy weight management by encouraging and supporting healthy eating behaviors, promoting a positive body image, and limiting negative weight talk. Whenever possible, obesity prevention and treatment interventions for adolescents should also involve the family and other supportive social networks.

## Competing interests

The authors declare that they have no competing interests.

## Authors’ contributions

VQ took the lead in writing the manuscript and in incorporating revisions from other authors. MW conducted the data analysis and assisted in data interpretation and manuscript writing. NL made contributions in the acquisition of data and revisions of the manuscript. JH contributed to the interpretation of data and made revisions to the manuscript. DNS is the Principal Investigator of the Project EAT study and contributed to revisions of the manuscript. All authors approved the final manuscript submission.
